# Novel *N,N*-Dimethyl-idarubicin Analogues Are Effective Cytotoxic Agents for ABCB1-Overexpressing, Doxorubicin-Resistant Cells

**DOI:** 10.1021/acs.jmedchem.4c00614

**Published:** 2024-08-01

**Authors:** Merle A. van Gelder, Yufeng Li, Dennis P. A. Wander, Ilana Berlin, Hermen S. Overkleeft, Sabina Y. van der Zanden, Jacques J. C. Neefjes

**Affiliations:** † Department of Cell and Chemical Biology, ONCODE Institute, Leiden University Medical Center, Einthovenweg 20, 2333 CZ Leiden, The Netherlands; ‡ Leiden Institute of Chemistry, Leiden University, Einsteinweg 55, 2333 CC Leiden, The Netherlands

## Abstract

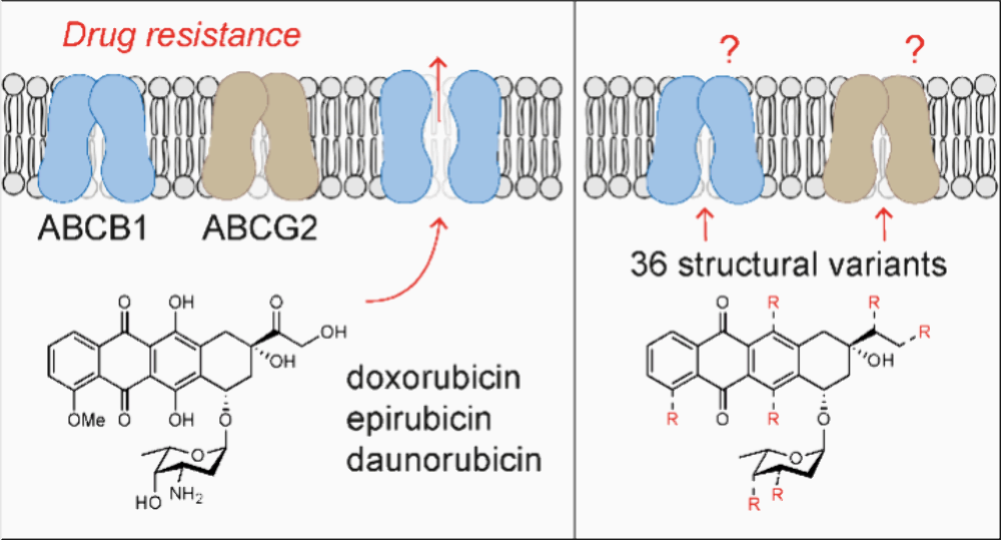

Anthracyclines comprise one of the most effective anticancer drug classes. Doxorubicin, daunorubicin, epirubicin, and idarubicin have been in clinical use for decades, but their application remains complicated by treatment-related toxicities and drug resistance. We previously demonstrated that the combination of DNA damage and histone eviction exerted by doxorubicin drives its associated adverse effects. However, whether the same properties dictate drug resistance is unclear. In the present study, we evaluate a library of 40 anthracyclines on their cytotoxicity, intracellular uptake, and subcellular localization in K562 wildtype versus ABCB1-transporter-overexpressing, doxorubicin-resistant cells. We identify several highly potent cytotoxic anthracyclines. Among these, *N,N*-dimethyl-idarubicin and anthracycline (composed of the idarubicin aglycon and the aclarubicin trisaccharide) stand out, due to their histone eviction-mediated cytotoxicity toward doxorubicin-resistant cells. Our findings thus uncover understudied anthracycline variants warranting further investigation in the quest for safer and more effective anticancer agents that circumvent cellular export by ABCB1.

## Introduction

Anthracyclines are among the most valuable anticancer drugs in current clinical use. They boast remarkably broad anticancer activity and have therefore become integral to the treatment of numerous tumor types.^1^ Despite their high effectivity, clinical application of anthracyclines is hindered by severe side effects as well as drug resistance.^2,3^ Recent years have witnessed key breakthroughs in our understanding of anthracycline efficacy and treatment-induced side effects.^4,5^ However, the relationship between anthracycline activity and drug resistance remains incompletely understood.

Doxorubicin and its close structural analogues epirubicin, daunorubicin, and idarubicin induce cell death through two main mechanisms of action: generation of DNA double-strand breaks (DNA damage) and eviction of histones from chromatin (chromatin damage).^6^ For decades, the therapeutic effects of these anthracyclines have been ascribed primarily to their DNA damaging activity. However, recent evidence revealed that the natural product anthracycline aclarubicin, as well as the synthetic doxorubicin analogue *N,N*-dimethyldoxorubicin, exclusively induce histone eviction.^7^ These anthracyclines are at least equally as effective against tumor cells as doxorubicin in murine *in vivo* models but much less toxic to healthy cells and tissues. For instance, *N,N*-dimethyldoxorubicin does not cause cardiotoxicity, secondary tumor formation, or gonadal dysfunction *in vivo*, unlike its parent compound doxorubicin.^5^ Along the same lines, clinical observations reveal that aclarubicin treatment is less cardiotoxic for cancer patients than doxorubicin treatment, while both compounds appear to be equally effective as anticancer agents.^8,9^ Importantly, clinical use of anthracyclines is not only limited by off-target toxicities but also hindered by the emergence of drug resistance. Drug resistance poses a critical barrier to treatment and remains one of the leading causes of chemotherapy failure in cancer patients.^10^ A key event in drug resistance is the upregulation of the ATP-binding cassette (ABC) transporters. Notable among these are ABCB1, also known as p-glycoprotein (p-gp),^11^ and ABCG2, also referred to as breast cancer resistance protein (BCRP).^12^ The ABC transporters are responsible for the efflux of a wide range of chemotherapeutics across the plasma membrane, leading to lower intracellular drug levels and treatment resistance. Doxorubicin and its clinically used analogues (epirubicin, daunorubicin, and idarubicin) all are known substrates for ABCB1,^13^ and several studies have noted increased ABCB1 expression in tumor cells in response to anthracycline chemotherapy.^14^

Ideally, new anthracycline leads for clinical development would address both of the limitations discussed above by eliciting less adverse events *and* circumventing molecular mechanisms that underlie drug resistance. Despite promising novel approaches in anthracycline synthesis, such as doxorubicin-conjugate structures and drug carriers, the development of more tolerable anthracyclines has proven difficult.^15,16^ In recent years, we have conducted structure–activity relationship (SAR) studies on doxorubicin/aclarubicin analogues, where we systematically varied the nature of the tetracycline aglycon, the sugar (unsubstituted or glycosylated 3-amino-2,3-dideoxy-l-fucose including configurational isomers), and the aminosugar N-alkylation pattern. This yielded a comprehensive anthracycline library of 40 entries that serves as the basis of the studies presented here. Biochemical evaluation of the above structural variants revealed a general trend indicating that aminosugar *N*-dimethylation enhances cytotoxicity *in vitro*. In addition, histone eviction capacity has proven to be more predictive of anthracycline cytotoxicity than their DNA damaging activity.^17^ In this study, we considered whether our current anthracycline collection contains novel anticancer agents that combine potent cancer cell killing with low susceptibility to export by ABC transporters. Previous research has suggested that alkylation of the sugar 3′-amino group in doxorubicin/aclarubicin-type anthracyclines yields compounds that operate exclusively through histone eviction.^7,17,18^ In addition, *N*-methylation of oxaunomycin variants increased their cytotoxicity toward drug-resistant cells.^19^ The importance of structural variations in the saccharide chains has also been emphasized in the context of drug resistance, since arimetamycin A hybrid structures with doxorubicin and daunorubicin maintained nanomolar activity against drug-resistant cells *in vitro*.^20^

Therefore, we now profiled our library of structural variants for their cytotoxicity in wildtype K562 leukemia cells versus those overexpressing ABCB1 or ABCG2. In general, we find that *N,N*-dimethylation renders an anthracycline a poor substrate for ABC transporters. We also compared intracellular anthracycline levels and their subcellular localization to evaluate whether a correlation exists between these factors and cytotoxicity. Our results indicate that intracellular anthracycline levels are not directly linked to the cytotoxicity of anthracyclines but that subcellular localization, and in particular nuclear concentration, correlates with cytotoxicity, both in the absence and in the presence of ABCB1 overexpression. Collectively, we identify derivatives of doxorubicin, epirubicin, and idarubicin with high effectivity against cancer cells, which have been rendered doxorubicin-resistant through overexpression of ABC transporters. Relevant structural changes did not reduce either the DNA intercalation affinity of the compounds or their targeting of Topoisomerase IIα (TopoIIα). Given that these derivatives specifically induce histone eviction without causing DNA double-strand breaks, they may also feature more favorable side-effect profiles as compared to their parent compounds.

## Results and Discussion

**Figure 1:**
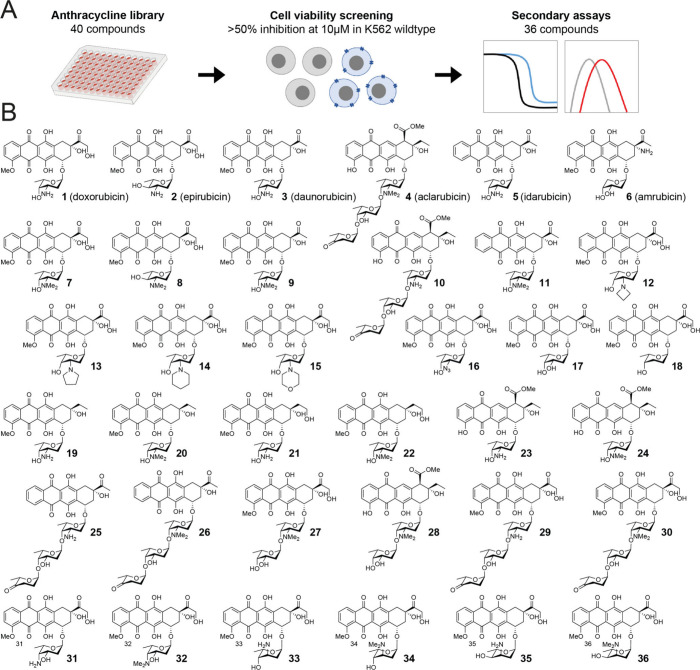
Workflow and chemical structures of compounds evaluated in this study. (A) Schematic overview of the threshold for inclusion of the 36 anthracycline variants used for cellular assays. (B) Chemical structures of compounds evaluated in all cellular assays. These contain the clinically used anthracyclines doxorubicin (**1**), epirubicin (**2**), daunorubicin (**3**), aclarubicin (**4**), and idarubicin (**5**), and synthetic anthracyclines (**6**–**36**).

Anthracyclines are commonly used in the treatment of hematological malignancies. To evaluate the effects of different anthracycline variants, we performed cytotoxicity profiling of 40 compounds (Figure S1) in the human myelogenous leukemia cell line K562. Of these, compounds **1**–**6** are in clinical use and commercially available, whereas synthesis of compounds **7**–**36** and **S1**–**S4** was reported previously.^7,17,18^ In total, 36 out of the 40 compounds passed the threshold of at least 50% growth inhibition at a 10 μM concentration (Figure 1A) and were selected for further evaluation.

### Cytotoxicity Profiling of Anthracycline Variants in ABCB1- and ABCG2-Mediated Drug Resistance

**Figure 2:**
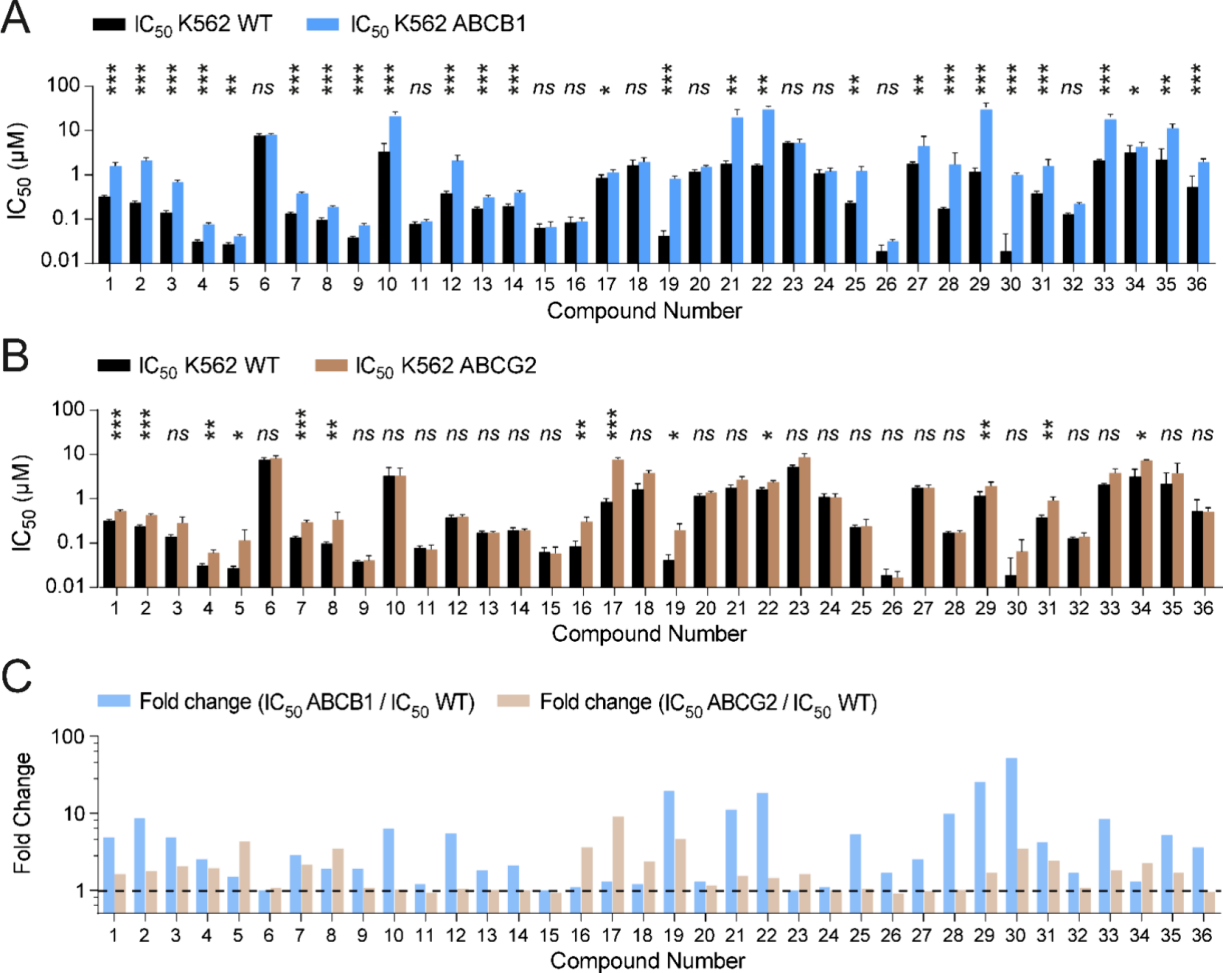
Cytotoxicity profiles of anthracyclines tested *in vitro*. (A) IC_50_ values are plotted for all compounds tested in wildtype K562 cells and ABCB1-overexpressing cells. (B) IC_50_ values are plotted for all compounds tested in wildtype K562 cells and ABCG2-overexpressing cells. Data is shown as mean ± SD. The *X*-axis shows the numbers of the compounds corresponding to Figure 1B. Two-way ANOVA; *p < 0.05; **p < 0.01; ***p < 0.001; ns, not significant. (C) Fold change difference between the IC_50_ value in ABCB1-overexpressing versus wildtype cells (IC_50_(ABCB1)/IC_50_(wildtype)) and fold change difference between the IC_50_ value in ABCG2-overexpressing versus wildtype cells (IC_50_(ABCG2)/IC_50_(wildtype)) is plotted for every compound. The dotted line indicates a fold change of 1, e.g., no difference in IC_50_.

First, cytotoxicity profiles of anthracycline variants (**1**–**36**, Figure 1B) in wildtype K562 cells were compared to those in cells overexpressing drug transporters ABCB1 or ABCG2 using a cell growth inhibition assay. In short, cells were treated for 2 h with different anthracycline variants, washed, and left to grow for 72 h. Subsequently, cell viability was measured relative to untreated control cells. IC_50_ values for all compounds were plotted for K562 wildtype cells versus ABCB1- (Figure 2A) or ABCG2- (Figure 2B) overexpressing cells; calculated IC_50_ values are included in Table S1. Direct comparisons between 36 compounds per cell line (Figure 2A, B) are complemented by relative (fold-change) analysis of cytotoxic effects of a single compound against wildtype versus ABCB1- or ABCG2-overexpressing cells (Figure 2C). Both parameters are relevant drug performance measures. Cytotoxicity, expressed as the IC_50_ value, should ideally be low in all cell lines for a compound to be, or become, an effective anticancer agent. Additionally, fold change in the IC_50_ value, IC_50_(ABCB1)/IC_50_(wildtype) or IC_50_(ABCG2)/IC_50_(wildtype), is indicative of transporter substrate status, providing critical SAR information on anthracycline variants as anticancer agents. In agreement with earlier findings on their susceptibility to ABCB1-mediated tumor resistance, anthracycline variants presently in clinical use (**1**–**4**) display poor cytotoxicity profiles against cancer cells overexpressing ABCB1, with 2.5- to 9-fold reduction in potency as compared to their respective cytotoxicity in wildtype cells. Idarubicin (**5**), on the other hand, was nearly equally toxic toward ABCB1-overexpressing as wildtype cells. The same trend of reduced cytotoxicity was observed when testing clinically used anthracyclines (**1**–**4**) against ABCG2-overexpressing cells, although in a much smaller magnitude. Idarubicin (**5**), however, was considerably less cytotoxic toward ABCG2-overexpressing cells compared to wildtype cells. Amrubicin (**6**) was poorly cytotoxic in all three cell lines, regardless of drug transporter overexpression. *N,N*-Dimethyldoxorubicin (**7**), *N,N*-dimethylepirubicin (**8**), and *N,N*-dimethyldaunorubicin (**9**) all proved to be much more cytotoxic against both ABCB1- and ABCG2-overexpressing cells when compared to their non-methylated counterparts (**1**–**3**). This relationship between the pattern of *N*-methylation and cytotoxicity was also apparent when aclarubicin (**4**) was compared to its primary amine counterpart (**10**). Interestingly, cytotoxicity of idarubicin (**5**) in wildtype cells was superior to that of *N,N*-dimethyl-idarubicin (**11**), but the fold change in IC_50_ (ABCB1/wildtype) and (ABCG2/wildtype) of compound **11** was negligible. Within the set of cyclic, tertiary amines, compounds **13**–**15** outperformed their parental drug doxorubicin in both wildtype and ABCB1-overexpressing cells, unlike azetidine (**12**). Compounds **12**–**15** were all equally cytotoxic against wildtype cells and ABCG2-overexpressing cells. Of the three doxorubicin derivates lacking a basic amine, variants **17** and **18** were considerably less cytotoxic than doxorubicin (**1**) in wildtype cells, but the fold change in IC_50_ (ABCB1/wildtype) was close to 1. Morpholino-doxorubicin (**15**) and azido-doxorubicin (**16**) proved to be the most effective derivatives of doxorubicin (**1**) in this set of compounds against wildtype and ABCB1-overexpressing cells. This is in line with previous studies reporting on cytotoxicity of 3′-azido and 3′-morpholinyl analogues of doxorubicin and daunorubicin in a treatment-induced drug-resistant K562 cell line.^21,22^ Yet, azido-doxorubicin (**16**) was markedly less cytotoxic against ABCG2-overexpressing cells. Removal of the aglycone carbonyl function, as in **19**–**22**, generally did not result in compounds with enhanced cytotoxicity to either wildtype or ABCB1-overexpressing cells when compared to their respective parent compounds (**1**, **3**). Compounds **23** and **24**, comprising hybrids between aclarubicin (aglycon part), doxorubicin, and *N,N*-dimethyldoxorubicin, also turned out to be weaker cytotoxic agents when compared to their parent compounds (**1**, **7**). However, they displayed no difference in cytotoxicity toward wildtype versus ABCB1-overexpressing cells. Cytotoxicity profiles in ABCG2-overexpressing cells followed the same trend for compounds **19**–**24**, though the observed fold changes IC_50_ (ABCG2/wildtype) were smaller in comparison. Of the idarubicin-aglycon-containing trisaccharides (**25** and **26**), the dimethylated variant (**26**) showed the best cytotoxicity among all 36 compounds, with an IC_50_ of 19 nM in wildtype cells and ABCG2-overexpressing cells, and 32 nM in ABCB1-overexpressing cells. Doxorubicin/aclarubicin hybrid structures **27**–**30** proved to be only modestly cytotoxic against ABCB1- and ABCG2-overexpressing cells, and of these compounds, only *N*-dimethyl variants **28** and **30** were potent killers of wildtype cells. Finally, among doxorubicin isomers **31**–**36**, only the regioisomer **32** outperformed doxorubicin (**1**) in killing wildtype, ABCB1-, or ABCG2-overexpressing cells.

**Figure 3:**
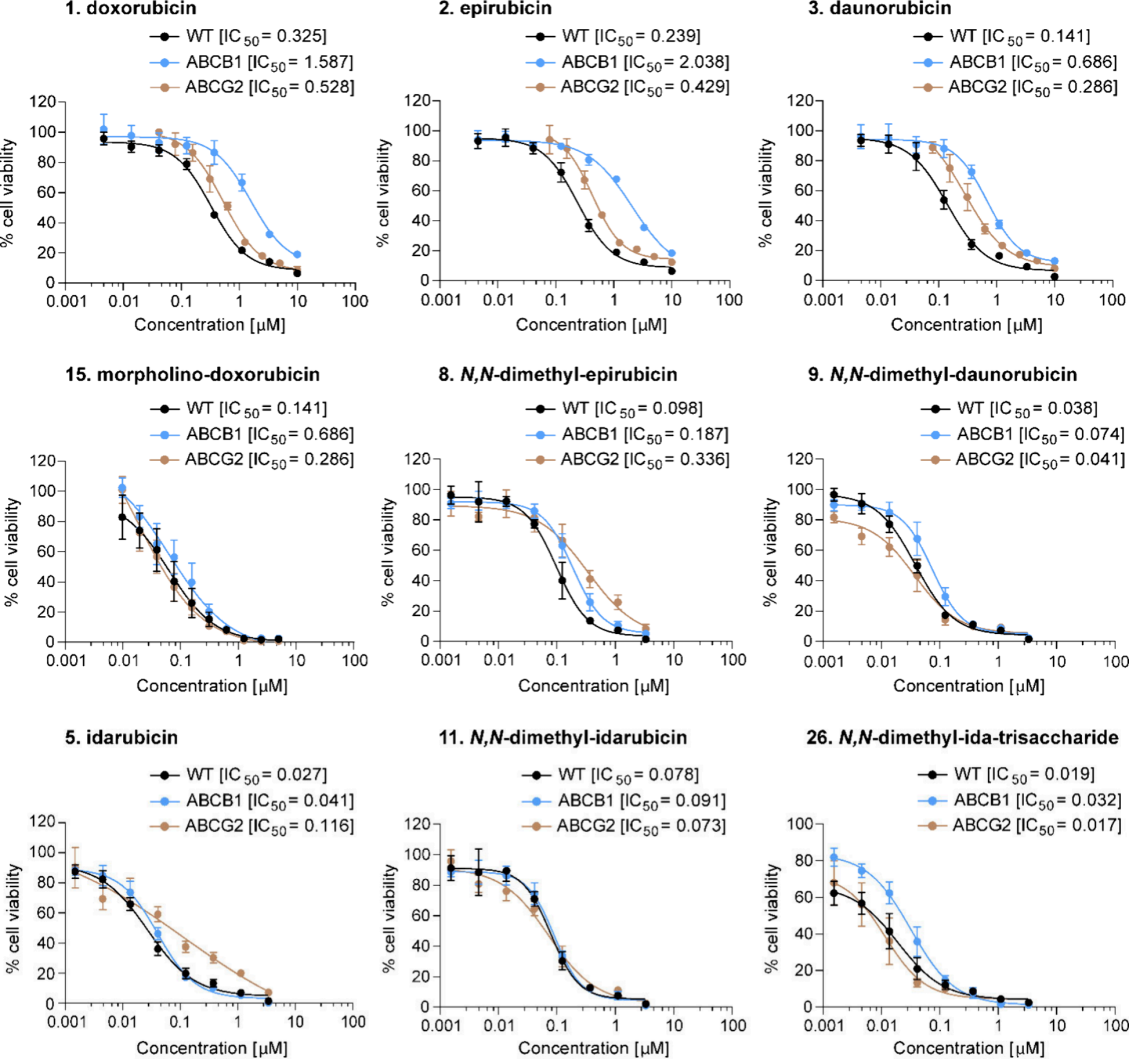
Individual IC_50_ curves plotted for the clinically used anthracyclines doxorubicin (**1**), epirubicin (**2**), daunorubicin (**3**), and idarubicin (**5**) and the most potent analogues of these anthracyclines in terms of IC_50_ and fold change IC_50_ between wildtype K562 cells and ABCB1- or ABCG2-overexpressing cells.

Examination of the fold change in IC_50_ (ABCB1/wildtype) (threshold set at 2) revealed 16 compounds in our library to be poor ABCB1 substrates, if at all (**5**, **6**, **8**, **9**, **11**, **13**, **15**–**18**, **20**, **23**, **24**, **26**, **32**, **34**). However, most anthracycline variants tested remained effective in ABCG2-overexpressing cells, where the cytotoxicity of 23 compounds (**3**, **6**, **9**–**15**, **18**, **20**, **21**, **23**–**28**, **30**, **32**, **33**, **35**, **36**) was not significantly altered compared to that of wildtype cells. The fold change differences between IC_50_ (ABCB1/wildtype) and IC_50_ (ABCG2/wildtype) show a similar trend, but the amplitude is much smaller in the latter case (Figure 2C). Since the clinical relevance of ABCG2 is less well established than that of ABCB1 in the context of anthracyclines,^23^ we continued with the ABCB1 transporter-mediated drug-resistant cells for further evaluation. From our complete library of anthracyclines, morpholino-doxorubicin (**15**), *N,N*-dimethyl-epirubicin (**8**), *N,N*-dimethyl-daunorubicin (**9**), and in particular *N,N*-dimethyl-idarubicin (**11**) and *N,N*-dimethyl-idarubicin-trisaccharide (**26**) stood out because of their low IC_50_ values in wildtype cells and ABCB1-overexpressing cells (Figure 3) and low (**8**, **9**, **15**) to negligible (**11**, **26**) relative fold change in IC_50_ (ABCB1/wildtype). The cytotoxic effects of these compounds (**8**, **9**, **11**, **15**, and **26**) were also confirmed in a live/dead assay (Figure S2). Altogether, the aglycon of either daunorubicin (**3**) or idarubicin (**5**), combined with aminosugar *N*-methylation, provided the most favorable structural elements for improved cytotoxicity profiles against wildtype as well as ABCB1-overexpressing cells.

### Intracellular Drug Accumulation Is Not a Proxy for Cytotoxicity

**Figure 4:**
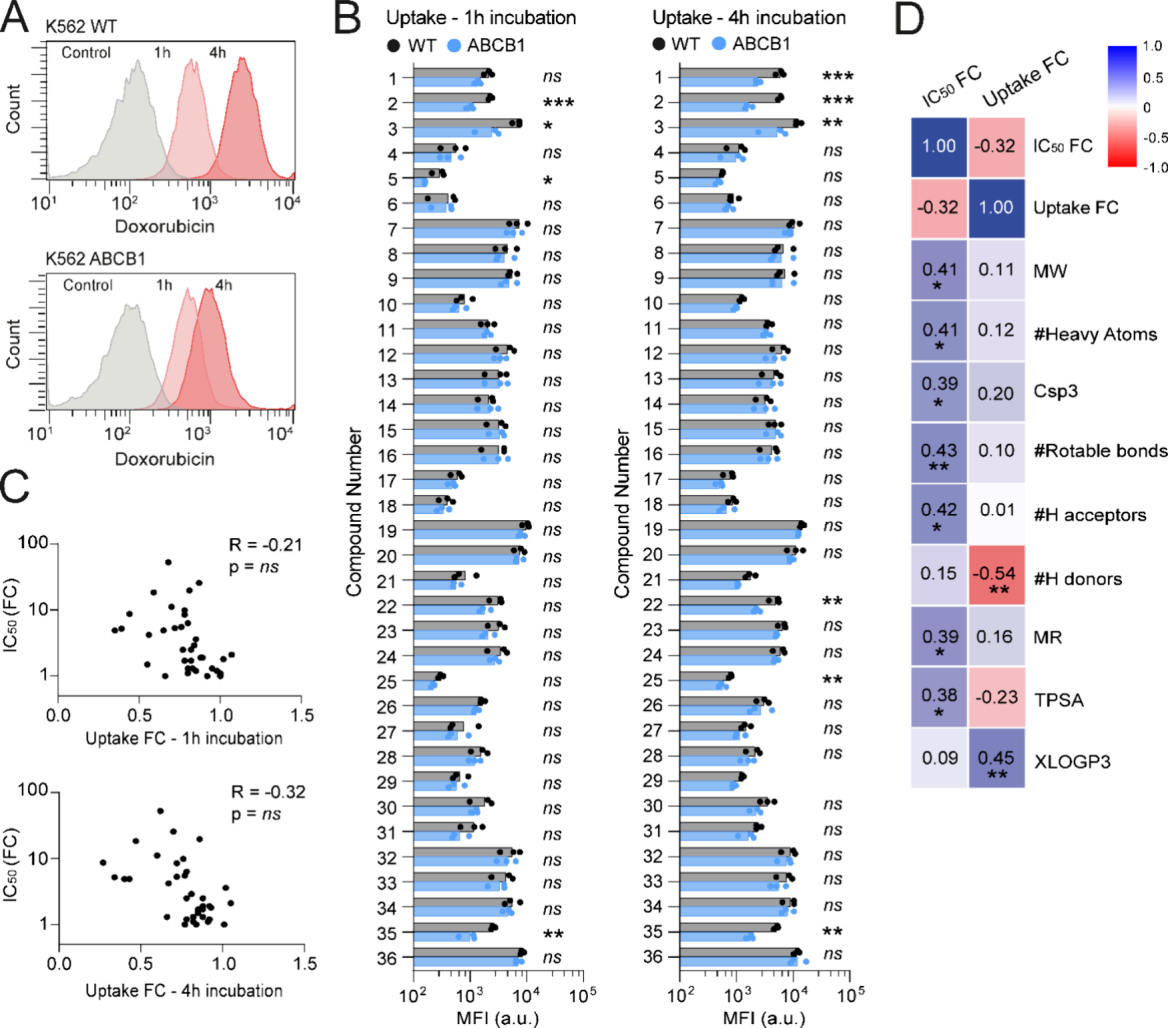
Intracellular fluorescence of all compounds 1 and 4 h post treatment. Mean fluorescent intensity was measured with flow-cytometry (A), and fluorescent intensity was compared 1 and 4 h post treatment (B) between K562 wildtype and ABCB1-overexpressing cells. (C) The fold change in intracellular fluorescence does not significantly correlate to the fold change IC_50_ between wildtype and ABCB1-overexpressing cells. (D) Pearson’s *r* correlation matrix displaying the correlation between computationally predicted biochemical parameters and fold change IC_50_ and fold change intracellular fluorescence. Positive correlations are displayed in blue, and negative correlations are in red. Color intensity is proportional to the correlation coefficients. MW = molecular weight; Csp3 = fraction of carbon atoms with sp^3^ hybridization; MR = molar refractivity; TPSA = topological polar surface area; XLOGP3 = LogP_o/w_.

All anthracycline variants **1**–**36** used in our study are fluorescent, allowing a comparison of their accumulation in cells using flow cytometry (Figure 4A). Both wildtype and ABCB1-overexpressing cells were exposed to each of the compounds, at a concentration of 10 μM, and intracellular fluorescence was measured after 1 or 4 h of incubation (Figure 4B). Following 1 h treatment, intracellular fluorescence of most compounds tested was similar between the cell lines, except for the clinically used variants epirubicin (**2**), daunorubicin (**3**), and idarubicin (**5**), as well as the doxorubicin stereoisomer (**35**). After 4 h of treatment, this trend of reduced intracellular fluorescence comparing wildtype versus ABCB1-overexpressing cells increased for compounds **1**–**3**, but not for idarubicin (**5**). In addition, a significant reduction in fluorescence was observed in ABCB1-overexpressing cells compared to wildtype cells for doxorubicin analogues **21**, **22**, and **35** and for idarubicin analogue **25**. Previously highlighted cytotoxic compounds in the ABCB1 background—morpholino-doxorubicin (**15**), dimethyl-epirubicin (**8**), dimethyl-daunorubicin (**9**), dimethyl-idarubicin (**11**), and idarubicin-trisaccharide (**26**)—all appear to accumulate equally well in wildtype and ABCB1-overexpressing cells, as concluded from their comparable intracellular fluorescence. Remarkably, analysis of the complete set of compounds revealed no significant relationship between the intracellular accumulation of compounds and their corresponding fold change in IC_50_ (ABCB1/wildtype) (Figure 4C). For instance, intracellular fluorescence of trisaccharide **30** was similar in wildtype and ABCB1-overexpressing cells, whereas its fold change in IC_50_ (ABCB1/wildtype) was nearly 50, indicating poor cytotoxicity in ABCB1-overexpressing cells, despite efficient intracellular accumulation. These results show that the extent of uptake does not solely define drug potency in the context of ABCB1-mediated drug resistance.

The ability of a drug to penetrate membranes is dependent on its various properties, which may, in turn, influence intracellular drug concentrations. To define these, we performed computational predictions of relevant biochemical, biophysical, and pharmacokinetic parameters for all 36 compounds (Tables S2 and S3), including molecular weight (MW), number of heavy atoms, saturation (fraction of sp^3^-hybridized carbons), flexibility (rotatable bonds), number of H-bond acceptors and donors, molar refractivity, polarity (topological polar surface area), and lipophilicity (LogP). Association between these properties and the observed fold change in IC_50_ (ABCB1/wildtype) as well as intracellular fluorescence (ABCB1/wildtype) is displayed in a correlation matrix (Figure 4D). This analysis revealed that the fold change in IC_50_ correlates to parameters describing the size and polarity of the compounds, whereas the fold change in cellular uptake most strongly correlates with predicted compound lipophilicity. Although the latter is in agreement with previous research on hydrophobic compounds as better substrates for ABCB1,^24^ we find that intracellular drug accumulation does not directly explain the observed differences in drug cytotoxicity profiles.

### Nuclear Uptake of Anthracyclines Is a Strong Predictor of Cytotoxicity

**Figure 5:**
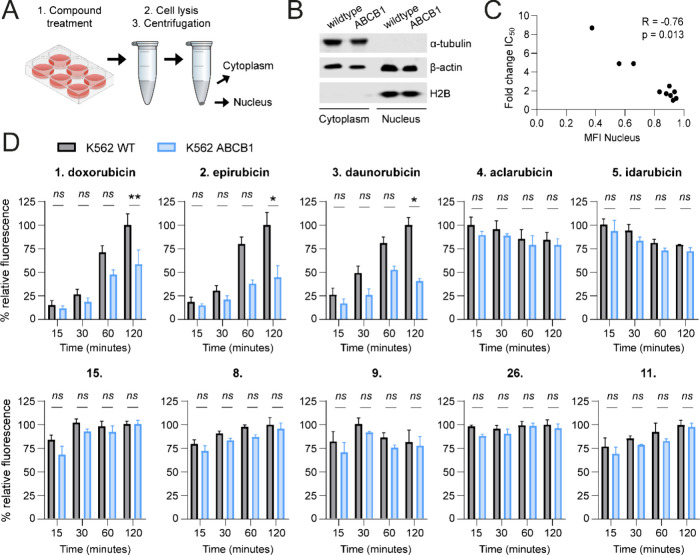
Nuclear accumulation of selected compounds **1**–**5**, **8**, **9**, **11**, **15**, and **26**. Numbers correspond to the structures in Figure 1. (A) K562 wildtype and ABCB1-overexpressing cells were treated with 10 μM of the indicated compounds and subjected to fractionation. (B) Separation of the fractions was confirmed with Western blot. (C) Correlation between fold change IC_50_ and mean fluorescence intensity in nuclear fraction. (D) Mean fluorescence intensity in the nuclear fraction was measured at a series of time points (15, 30, 60, and 120 min). Fluorescence was normalized to the largest signal. Two-way ANOVA; *p < 0.05; **p < 0.01; ***p < 0.001; ns, not significant.

Since intracellular anthracycline accumulation did not directly reflect drug potency in killing ABCB1-overexpressing cells, we hypothesized that nuclear tarting may provide a better measure in this regard. To address this, we set out to determine the subcellular localization of clinical anthracyclines (**1**–**5**) and their most potent analogues (**8**, **9**, **11**, **15**, and **26**) identified in our cytotoxicity assays (Figure 2). To this end, K562 wildtype and ABCB1-overexpressing cells were treated for a series of time points with each anthracycline, lysed, and subjected to fractionation (Figure 5A and 5B). Fluorescence in cytoplasmic and nuclear fractions was then measured with a plate reader. Nuclear accumulation of doxorubicin (**1**), epirubicin (**2**), and daunorubicin (**3**) was significantly decreased after 1 and 2 h treatment in ABCB1-overexpressing cells compared to wildtype cells. On the other hand, aclarubicin (**4**) and idarubicin (**5**) showed comparable nuclear fluorescence in both cell types (Figure 5D). Compounds **8**, **9**, and **15** exhibited superior nuclear accumulation over time compared to their respective clinically used counterparts (**1**, **2** and **3**). For these compounds, as well as compounds **11** and **26**, no significant difference was observed in nuclear fluorescence between wildtype and ABCB1-overexpressing cells. Taken together, nuclear compound fluorescence correlated significantly with the fold change in IC_50_ (ABCB1/wildtype) (Figure 5C). The difference in cytoplasmic accumulation between wildtype cells and ABCB1-overexpressing cells was less substantial (Figure S3A), and the observed cytoplasmic fluorescence was less indicative of the change in cytotoxicity (Figure S3B). To test whether subcellular localization was influenced by ABCB1 activity, we pretreated ABCB1-overexpressing cells with the ABCB1 inhibitor Tariquidar (TRQ) prior to anthracycline treatment and subsequent fractionation analysis. Both cytoplasmic and nuclear fluorescence could be restored to levels similar to those observed in wildtype cells, as demonstrated in Figure S4 for doxorubicin (**1**) and daunorubicin (**3**). No effect of ABCB1 inhibition was observed for idarubicin (**5**) or compounds **9**, **11**, and **15**. This suggests that superior nuclear accumulation of compounds **5**, **9**, **11**, and **15** is independent of ABCB1 expression and that nuclear accumulation is a strong predictor of anthracycline effectivity. Targeting of TopoIIα in the nucleus was also assessed upon treatment with all compounds (**1**–**36**) using confocal time-lapse microscopy (Table S1 and Figure S5). As expected, treatment with the clinically relevant anthracyclines (**1**–**5**) and their most potent analogues (**8**, **9**, **11**, **15**, and **26**) was found to cause marked redistribution of nuclear TopoIIα.

**Figure 6:**
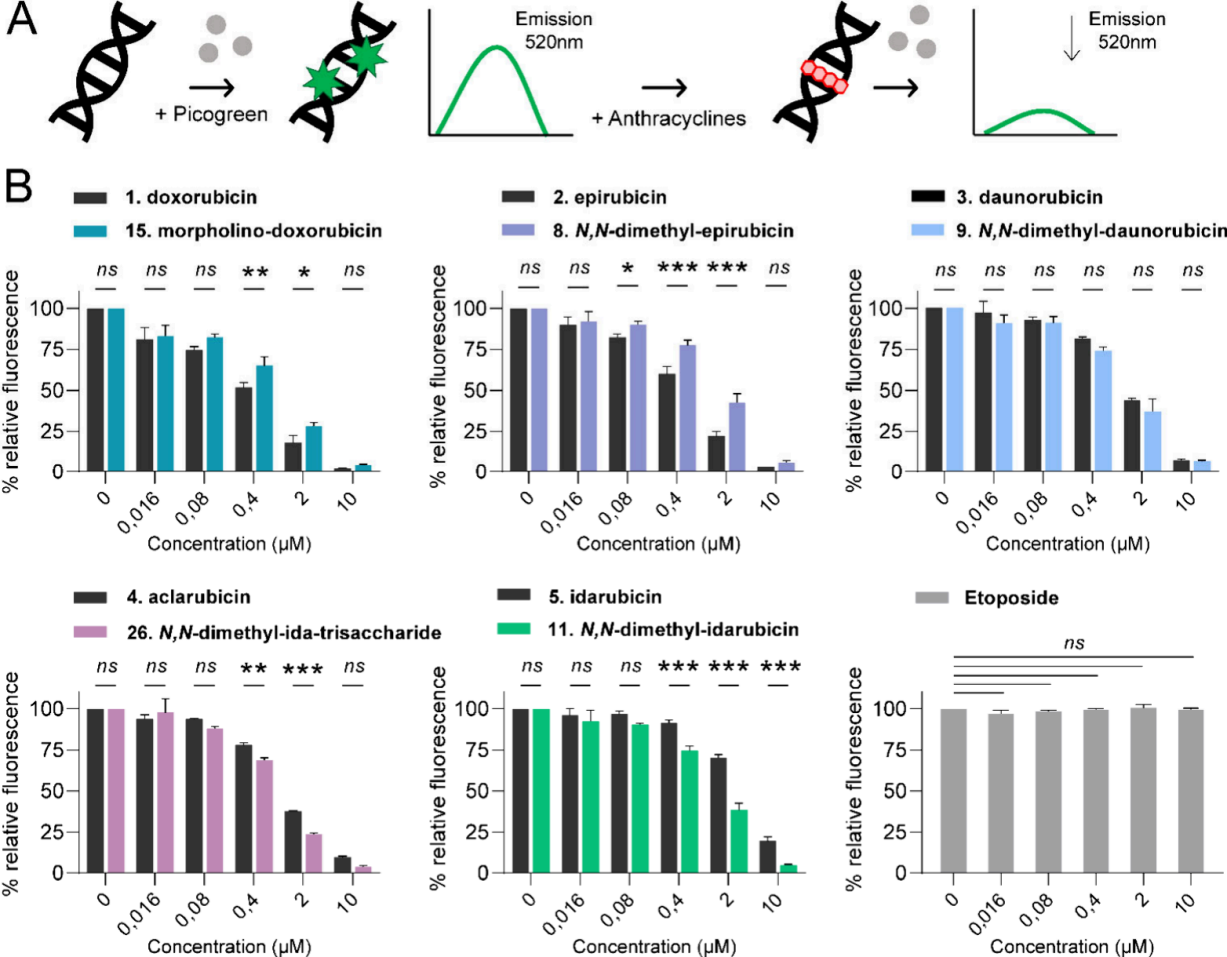
DNA intercalation affinity of selected compounds **1**–**5**, **8**, **9**, **11**, **15**, and **26**. Numbers correspond to the structures in Figure 1. (A) The intercalation of anthracyclines into double-stranded DNA was tested with a competition dye displacement assay. (B) The percentage of initial fluorescence is plotted against the concentrations of the indicated compounds. Two-way ANOVA; *p < 0.05; **p < 0.01; ***p < 0.001; ns, not significant.

Further, the intercalation of anthracyclines into double-stranded DNA was tested with a competition dye displacement assay. DNA intercalation affinity of clinical anthracyclines (**1**–**5**) was compared to that of their most potent analogues (**8**, **9**, **11**, **15**, and **26**). Intercalation was determined using PicoGreen, which becomes fluorescent upon binding to double-stranded (ds) DNA, and addition of compounds displacing intercalated PicoGreen thus results in the loss of fluorescence signal (Figure 6A). Plotting remaining fluorescence against compound concentration (Figure 6B) showed that doxorubicin (**1**) and epirubicin (**2**) share higher DNA binding affinity at lower concentrations compared to morpholino-doxorubicin (**15**) and *N,N*-dimethyl-epirubicin (**9**), respectively. Daunorubicin (**3**) and *N,N*-dimethyl-daunorubicin (**10**) were equally effective, and *N,N*-dimethyl-idarubicin (**11**) and *N,N*-dimethyl-idarubicin-trisaccharide (**26**) proved to be particularly efficient intercalating agents. The non-intercalating Topo II poison, etoposide, was used as a negative control. Ultimately, structural variations of our lead compounds did not alter their canonical anthracycline functions of DNA intercalation and TopoIIα targeting.

## Conclusion

Anthracyclines doxorubicin (**1**), daunorubicin (**2**), and epirubicin (**3**) have been in clinical use for decades. However, their effectivity is severely limited by both treatment-related toxicities and drug resistance. Preferably, anthracyclines of the future should address both of these issues to cause fewer adverse events while retaining potency against drug-resistant cells. In previous studies, we have shown that the archetypal anthracycline, doxorubicin (**1**), as well as its close structural analogues **2** and **3**, exert their anticancer activity through two independent mechanisms: induction of DNA double-strand breaks and chromatin damage through histone eviction. Uncoupling these activities identified *N,N*-dimethyldoxorubicin (**7**) as an efficient histone-evicting compound that lacks the capacity for DNA double-strand break formation and also lacks most of the therapy-related side effects associated with the clinically used anthracyclines. In subsequent studies, we observed that anthracyclines containing an *N,N*-dimethyl aminosugar often harbor this desirable set of properties: they exert their activity exclusively through histone eviction and are generally more cytotoxic to tumor cells than their parent compound. At the same time, they display limited toxicity in healthy cells and tissues. We thus have amassed a set of anthracyclines with potential clinical value, alongside their relevant structural analogues as controls, namely, the 40 compounds used in this study. With their cytotoxicity profiles against tumor cells in hand and their mode(s) of action known, we investigated the second important parameter, which determines clinical applicability of new anthracyclines. Namely, performance in a doxorubicin-resistant tumor setting caused by the overexpression of drug exporter ABCB1.

Side-by-side comparison of cytotoxicity profiles of the 36 most cytotoxic compounds in K562 wildtype versus ABCB1-overexpressing cells revealed that the most widely used clinical variants (**1**–**3**) are much less effective in ABCB1-overexpressing cells. This is in agreement with these variants being substrates for ABCB1, and that drug resistance after treatment with these drugs can emerge in the clinic.^13^ In total, 16 compounds had a fold change in IC_50_ (ABCB1/wildtype) below 2, indicating that these variants retain their cytotoxicity in ABCB1-mediated drug-resistant cells. The cytotoxicity of all anthracyclines tested was less affected by ABCG2-mediated drug export. The transporters have overlapping substrate specificities to some extent but most importantly in transporting the anthracyclines currently in clinical use (**1**–**5**). Our study reveals that it is possible to design anthracyclines that defy ABCB1- and ABCG2-mediated efflux. This is important because, despite many efforts, blocking these transporters by small-molecule inhibitors has thus far not resulted in improved tumor responses in clinical studies.^23,25^ Therefore, anthracyclines, which are both potent killers of tumor cells and insensitive to ABC transporter-mediated export, may be attractive alternatives, especially if novel variants are less toxic to healthy tissues. In this respect, we observe that anthracyclines featuring an *N,N*-dimethyl aminosugar in general are poor substrates for the ABCB1 drug transporter as compared to their non-alkylated counterparts. This is of interest because the same modification also allows discrimination between the different mechanisms of action: DNA double-strand break generation versus histone eviction. In addition, we show that nuclear accumulation is the strongest predictor of anthracycline effectivity in ABCB1-overexpressing, drug-resistant cells, and the most potent analogues identified in this study show improved nuclear accumulation compared with their clinically used counterparts.

Combining these new structure–activity insights into ABC transporter-mediated drug resistance with our earlier findings, we see the emergence of a new set of promising anthracyclines for further (pre)clinical development. The most potent structural variants of clinically used anthracyclines identified in this study are morpholino-doxorubicin (**15**), *N,N*-dimethyl-epirubicin (**8**), *N,N-*dimethyl-daunorubicin (**9**), *N,N*-dimethyl-idarubicin (**11**), and trisaccharidic *N,N*-dimethyl-idarubicin (**26**). We previously showed that these compounds are among the most potent histone evictors and do not induce DNA double-strand breaks.^17^ We now find that these variants are more cytotoxic toward both wildtype and ABCB1- or ABCG2-overexpressing cells than their parent compounds. By this virtue, we expect that these compounds are superior in circumventing ABCB1-mediated resistance, a significant type of resistance to doxorubicin and its structural analogues. Moreover, because none of these compounds cause DNA damage, there is also less risk of acquired resistance through increased DNA damage repair or decreased Topoisomerase II expression, a second mechanism of drug resistance observed in doxorubicin-resistant cells.^26,27^

In conclusion, this study contributes to the design and identification of new, more effective, and more benign anthracycline drugs. We performed several structure–activity analyses, which may help in defining design parameters for potentially successful new anthracyclines. Previously, we unearthed structural elements that determine DNA damage and histone eviction and showed that the combination of these activities is at the root of many side effects. Here, we add structure–activity information on anthracyclines in a drug resistance context by defining structural elements that enable circumventing ABCB1-mediated export and lead to enhanced nuclear accumulation. The most promising compounds presented here, and in particular idarubicin derivatives **11** and **26**, may deserve further exploration in preclinical studies, for instance to explore their effectivity in more advanced drug resistance models as well as to evaluate their *in vivo* efficacy.

## Experimental Section

The anthracycline variants **7**–**36** were synthesized as described previously and the compounds are >95% pure by HPLC analysis.^7,17,18^

### Reagents and Antibodies

Doxorubicin and epirubicin were obtained from Accord Healthcare Limited, UK, daunorubicin was obtained from Sanofi, and aclarubicin (sc-200160), idarubicin (sc-204774), and amrubicin (sc-207289) were purchased from Santa Cruz Biotechnology (USA). Tariquidar (SML1790) was purchased from Sigma-Aldrich (USA). Primary antibodies used for Western blotting: ABCB1 (1:1000, 13342, Cell Signaling), Histone H2B (1:1000, 12364, Cell Signaling), β-actin (1:10000, A5441, Sigma), and PARP (1:1000, 9542, Cell Signaling). Secondary antibodies used for blotting: IRDye 800CW goat anti-mouse IgG (H+L) (926-32210, Li-COR, 1:10000), IRDye 800CW goat anti-rabbit IgG (H+L) (926-32211, Li-COR, 1:5000).

### Cell Culture

K562 cells (B. Pang, Leiden University Medical Center, The Netherlands) were maintained in RPMI-1640 medium supplemented with 8% FCS. K562 ABCB1 overexpression cells were generated as described^28^ and maintained in RPMI-1640 medium supplemented with 8% FCS. K562 ABCG2 overexpression cells were generated with single-guide RNAs targeting the promoter region of ABCG2 (Forward: CAC CGT GCC GCG CTG AGC CGC CAGC; Reverse: AAA CGC TGG CGG CTC AGC GCG GCAC). The guide RNA sequence was cloned into lentiSAMv2-Puro plasmid containing the gRNA scaffold and dCas9 sequence, and lentivirus was made as previously described.^28^ K562/SAM stable cells were transduced with virus containing the respective guide RNAs and then selected using puromycin (2 μg mL^–1^). Single clones of cells were picked and verified by using PCR and Sanger sequencing. K562 ABCB2 overexpression cells were maintained in RPMI-1640 medium supplemented with 8% FCS. All cell lines were maintained in a humidified atmosphere of 5% CO_2_ at 37 °C, regularly tested for the absence of mycoplasma, and the origin of cell lines was validated using STR analysis.

### Cell Viability Assay

Cells were seeded into a 96-well format (2000 cells/well). 24 h after seeding, cells were treated with indicated compounds for 2 h at various concentrations. Subsequently, the compounds were removed by washing and the cells were left to grow for an additional 72 h. Cell viability was measured using the CellTiter-Blue viability assay (Promega). Relative survival was normalized to untreated control cells and corrected for the background signal.

### Flow Cytometry

Cells were treated with 10 μM compound for the indicated time points. Samples were washed with PBS, collected, and fixed with paraformaldehyde. Live/dead staining was performed with DAPI (1:1000). Samples were analyzed by flow cytometry using BD FACS Aria II, with 561-nm laser and 610/20-nm detector. The cellular uptake of anthracyclines and the live/dead ratio were quantified using FlowJo software v10.10.

### ADME

The computation of physicochemical descriptors and prediction of ADME parameters was performed with the freely accessible webtool SwissADME.^29^

### Subcellular Fractionation

Cells were treated for a series of time points (15, 30, 60, and 120 min) with 10 μM of the indicated compounds. Cells were washed and lysed directly in lysis buffer (50 mM Tris·HCl pH 8.0, 150 mM NaCl, 5 mM MgCl_2_, 0.5% Nonidet P-40, 2.5% glycerol supplemented with protease inhibitors), collected, vortexed, and incubated on ice for 10 min. To collect the cytoplasmic fraction, samples were centrifuged for 10 min, 15,000×g at 4 °C. Both nuclear (pellet) and cytoplasmic (supernatant) fractions were washed, and fluorescence was measured with a plate reader (500-15 excitation/580-30 emission).

### Western Blot

Cellular fractions were lysed directly in SDS-sample buffer (2%SDS, 10% glycerol, 5% β-mercaptoethanol, 60 mM Tris-HCl pH 6.8, and 0.01% bromophenol blue). Samples were separated by SDS-PAGE and transferred to a nitrocellulose membrane. Blocking of the filters and antibody incubations were done in PBS supplemented with 0.1% (v/v) Tween and 5% (w/v) milk powder (skim milk powder, LP0031, Oxiod). Blots were imaged by an Odyssey Classic imager (Li-Cor).

### DNA Dye Competition Assay

1 μg/mL circular double-stranded DNA was incubated with Quant-iT PicoGreen dsDNA reagent (Termo Fisher Scientific P7581) for 5 min at RT. Subsequently, indicated drug concentrations were added to the DNA/PicoGreen reaction and incubated for another 5 min at RT followed by measurement of the PicoGreen fluorescence using a CLARIOstar plate reader (BMG Labtech) excitation 480 nm/emission 520 nm (480-20/520-10 filter). The fluorescence was quantified relative to that of the untreated controls. Fluorescent signals of all samples were corrected for the corresponding drug concentrations in the absence of DNA.

### Quantification and Statistical Analysis

Each experiment was performed in triplicate, unless stated otherwise. Error bars denote ± SD. Statistical analyses were performed using Prism 8 software (GraphPad Inc.). ns, not significant, *p < 0.05, **p < 0.01, ***p < 0.001.

## Supporting Information Available

Table S1, biological data; Table S2, ADME parameters; Table S3, computational predictions of pharmacokinetics parameters; Figure S1, chemical structures of complete compound library; Figure S2, cell death assays for selected compounds; Figure S3, cytoplasmic accumulation of selected compounds; Figure S4, subcellular accumulation in context of ABCB1 inhibition; Figure S5, TopoIIα imaging for all compounds; Figures S6 and S7, HPLC traces for lead compounds (PDF)
Molecular formula strings and tabulated biological assays data (CSV)

## Abbreviations Used

ABCB1: ATP binding cassette subfamily B member 1
ABCG2: ATP-binding cassette superfamily G member 2
TopoIIα: Topoisomerase IIα
TRQ: Tariquidar
