# Supplementary material for: Novel N,N-Dimethyl-idarubicin Analogues Are Effective Cytotoxic Agents for ABCB1-Overexpressing, Doxorubicin-Resistant Cells
Source: J Med Chem. 2024 Aug 1;67(16):13802–12. doi: 10.1021/acs.jmedchem.4c00614 (PMC11345819; doi:10.1021/acs.jmedchem.4c00614)
Supplement: Supplementary file 1 — jm4c00614_si_002.pdf [file jm4c00614_si_002.pdf]

Supporting information for:

Novel *N,N*-dimethyl-idarubicin analogues are effective cytotoxic agents for ABCB1-overexpressing, doxorubicin-resistant cells

Merle A. van Gelder[1], Yufeng Li[1], Dennis P.A. Wander[1,2], Ilana Berlin[1], Hermen S. Overkleeft[2], Sabina Y. van der Zanden[1,*], Jacques J.C. Neefjes[1,*]

[1] Department of Cell and Chemical Biology, ONCODE Institute, Leiden University Medical Center, Einthovenweg 20, 2333 CZ Leiden, The Netherlands

[2] Leiden Institute of Chemistry, Leiden University, Einsteinweg 55, 2333 CC Leiden, The Netherlands

* Corresponding authors

Sabina. Y. van der Zanden: s.y.van_der_zanden@lumc.nl
Jacques J.C. Neefjes: j.j.c.neefjes@lumc.nl

Table of Contents

A: Supplemental Tables

Table S1 – Biological data for compounds (**1-36**) S3
Table S2 – ADME parameters for compounds (**1-36**) S4
Table S3 - Computational predictions of pharmacokinetics parameters for compounds (**1-36**) S5

B: Supplemental Figures

Figure S1 – Chemical structures of complete compound library (**1-36**, **S1-S4**) S6
Figure S2 – Cell death assays for selected compounds (**1-5**, **8**, **9**, **11**, **15**, **26**) S7
Figure S3 – Cytoplasmic accumulation of selected compounds (**1-5**, **8**, **9**, **11**, **15**, **26**) S8
Figure S4 – Subcellular localization in context of ABCB1-inhibition S9
Figure S5 – TopoIIα imaging analysis for all compounds (**1-36**) S10

C: HPLC traces

Figure S6 – Purity analysis of compound **11** S14
Figure S7 – Purity analysis of compound **26** S15

A: Supplemental Tables

#	IC_{50} WT (µM)	IC_{50} ABCB1 (µM)	IC_{50} ABCG2 (µM)	Topo IIα targeting	DNA damage	Histone eviction
1	0,325	1,587	0,528	Yes	Yes ^[7]	Yes ^[7]
2	0,239	2,083	0,428	Yes	Yes ^[18]	Yes ^[18]
3	0,141	0,686	0,286	Yes	Yes ^[17]	Yes ^[17]
4	0,031	0,077	0,060	Yes	No ^[7]	Yes ^[7]
5	0,027	0,041	0,116	Yes	Yes ^[17]	Yes ^[17]
6	7,696	8,068	8,199	No	Yes ^[17]	No ^[17]
7	0,136	0,390	0,293	Yes	No ^[7]	Yes ^[7]
8	0,098	0,187	0,336	Yes	No ^[18]	Yes ^[18]
9	0,038	0,074	0,041	Yes	No ^[17]	Yes ^[17]
10	3,324	20,905	3,379	Yes	No ^[7]	No ^[7]
11	0,078	0,091	0,073	Yes	No ^[17]	Yes ^[17]
12	0,379	2,087	0,396	Yes	No ^[17]	Yes ^[17]
13	0,172	0,312	0,173	Yes	No ^[17]	Yes ^[17]
14	0,195	0,402	0,193	Yes	No ^[17]	Yes ^[17]
15	0,064	0,066	0,059	Yes	No ^[17]	Yes ^[17]
16	0,084	0,089	0,303	Yes	Yes ^[17]	No ^[17]
17	0,856	1,127	7,799	Yes	Yes ^[17]	No ^[17]
18	1,621	1,978	3,827	Yes	Yes ^[17]	No ^[17]
19	0,042	0,829	0,196	Yes	Yes ^[17]	No ^[17]
20	1,18	1,540	1,368	Yes	No ^[17]	Yes ^[17]
21	1,788	19,820	2,736	Yes	Yes ^[17]	No ^[17]
22	1,623	29,892	2,341	Yes	No ^[17]	Yes ^[17]
23	5,32	5,413	8,695	Yes	No ^[17]	Yes ^[17]
24	1,098	1,213	1,091	Yes	No ^[17]	Yes ^[17]
25	0,23	1,221	0,242	Yes	Yes ^[17]	Yes ^[17]
26	0,019	0,032	0,017	Yes	No ^[17]	Yes ^[17]
27	1,796	4,511	1,752	Yes	No ^[7]	Yes ^[7]
28	0,174	1,715	0,175	Yes	No ^[7]	Yes ^[7]
29	1,173	30,123	1,957	Yes	Yes ^[7]	No ^[7]
30	0,019	1,000	0,065	Yes	No ^[7]	Yes ^[7]
31	0,381	1,588	0,914	Yes	Yes ^[17]	Yes ^[17]
32	0,129	0,220	0,139	Yes	No ^[17]	Yes ^[17]
33	2,117	17,975	3,861	Yes	Yes ^[18]	No ^[18]
34	3,244	4,377	7,325	Yes	Yes ^[18]	Yes ^[18]
35	2,206	11,423	3,729	Yes	Yes ^[18]	Yes ^[18]
36	0,535	1,928	0,510	Yes	Yes ^[18]	Yes ^[18]
S1	>10	-	-	-	No	No
S2	>10	-	-	-	No	Yes
S3	>10	-	-	-	No	No
S4	>10	-	-	-	No	Yes

Table S1 – Compound numbers shown in column one correspond to the structure of Figure S1. Cytotoxicity (IC_{50} values) towards K562 wildtype, ABCB1 overexpressing and ABCG2 overexpressing cells were calculated for all compounds. Targeting of TopoIIα was examined after treatment with 10 µM of compounds. Individual images are depicted in Figure S4. DNA damage and histone eviction data was reported on before, data indicated with ^[7] was reproduced from reference #7, copyright 2020 American Chemical Society, data indicated with ^[18] was reproduced from reference #18, copyright 2021 American Chemical Society, data indicated with ^[17] was reproduced from reference #17, copyright 2023 American Chemical Society.'

#	MW	Heavy atoms	Fraction Csp3	#Rotatable bonds	H-bond acceptors	H-bond donors	MR	TPSA	XLOGP3	LogS
1	543,5	39	0,44	5	12	6	132,7	206,1	1,27	-3,46
2	543,5	39	0,44	5	12	6	132,7	206,1	1,27	-3,46
3	527,5	38	0,44	4	11	5	131,5	185,8	1,83	-4,04
4	811,9	58	0,62	10	16	4	203,5	217,1	3,8	-5,29
5	497,5	36	0,42	3	10	5	125,0	176,6	1,86	-3,95
6	483,5	35	0,4	3	10	5	120,2	176,6	0,92	-3,81
7	571,6	41	0,48	6	12	5	142,5	183,3	2,25	-3,9
8	571,6	41	0,48	6	12	5	142,5	183,3	2,25	-3,9
9	555,6	40	0,48	5	11	4	141,3	163,1	2,81	-4,47
10	783,8	56	0,6	9	16	5	193,7	239,8	2,82	-4,87
11	525,6	38	0,46	4	10	4	134,8	153,8	2,84	-4,38
12	583,6	42	0,5	6	12	5	149,1	183,3	2,23	-3,94
13	597,6	43	0,52	6	12	5	153,9	183,3	2,59	-4,2
14	611,6	44	0,53	6	12	5	158,7	183,3	2,95	-4,46
15	613,6	44	0,52	6	13	5	155.0	192,5	1,73	-3,92
16	569,5	41	0,44	6	14	5	134,8	229,8	3,08	-3,93
17	544,5	39	0,44	5	12	6	131,1	200,3	1,53	-3,25
18	528,5	38	0,44	5	11	5	130.0	180,1	2,5	-4,07
19	513,5	37	0,48	4	10	5	131,3	168,8	2,57	-4,51
20	541,6	39	0,52	5	10	4	141,1	146.0	3,55	-4,95
21	529,5	38	0,48	5	11	6	132,5	189.0	1,9	-3,94
22	557,6	40	0,52	6	11	5	142,3	166,2	2,88	-4,37
23	557,6	40	0,46	5	12	6	137,7	206,1	2,28	-3,6
24	585,6	42	0,5	6	12	5	147,5	183,3	3,26	-4,04
25	739,8	53	0,58	7	15	5	183.0	230,6	2,6	-4,64
26	767,8	55	0,6	8	15	4	192,8	207,8	3,07	-5,06
27	701,7	50	0,57	8	15	6	172,5	222.0	2,13	-4,2
28	699,7	50	0,58	8	14	5	175,6	201,8	2,94	-4,63
29	785,8	56	0,59	9	17	6	190,7	260,1	2,01	-3,05
30	813,8	58	0,61	10	17	5	200,5	237,3	2,99	-3,78
31	543,5	39	0,44	5	12	6	132,7	206,1	1,27	-3,48
32	571,6	41	0,48	6	12	5	142,5	183,3	2,25	-4,21
33	543,5	39	0,44	5	12	6	132,7	206,1	1,27	-4,13
34	571,6	41	0,48	6	12	5	142,5	183,3	2,25	-4,56
35	543,5	39	0,44	5	12	6	132,7	206,1	1,27	-3,46
36	571,6	41	0,48	6	12	5	142,5	183,3	2,25	-3,9

Table S2 – Computational predictions of physicochemical parameters. The prediction of ADME parameters was performed with the freely accessible webtool SwissADME.[24]

#	GI absorption	BBB permeant	ABCB1 substrate	CYP1A2 inhibitor	CYP2C19 inhibitor	CYP2C9 inhibitor	CYP2D6 inhibitor	CYP3A4 inhibitor	log Kp (cm/s)	Bio-availability score
1	Low	No	Yes	No	No	No	No	No	-8,71	0,17
2	Low	No	Yes	No	No	No	No	No	-8,71	0,17
3	Low	No	Yes	No	No	No	No	No	-8,22	0,17
4	Low	No	Yes	No	No	No	No	No	-8,55	0,17
5	Low	No	Yes	No	No	No	No	No	-8,01	0,55
6	Low	No	Yes	No	No	No	No	No	-8,6	0,55
7	Low	No	Yes	No	No	No	No	No	-8,19	0,17
8	Low	No	Yes	No	No	No	No	No	-8,19	0,17
9	Low	No	Yes	No	No	No	No	No	-7,69	0,17
10	Low	No	Yes	No	No	No	No	No	-9,08	0,17
11	Low	No	Yes	No	No	No	No	No	-7,49	0,55
12	Low	No	No	No	No	No	No	No	-8,28	0,17
13	Low	No	No	No	No	No	No	No	-8,11	0,17
14	Low	No	No	No	No	No	No	No	-7,94	0,17
15	Low	No	No	No	No	No	No	No	-8,81	0,17
16	Low	No	Yes	No	No	No	No	No	-7,59	0,17
17	Low	No	Yes	No	No	No	No	No	-8,54	0,17
18	Low	No	Yes	No	No	No	No	No	-7,75	0,17
19	Low	No	Yes	No	No	No	No	No	-7,61	0,55
20	Low	No	Yes	No	No	No	No	No	-7,08	0,55
21	Low	No	Yes	No	No	No	No	No	-8,18	0,17
22	Low	No	Yes	No	No	No	No	No	-7,66	0,17
23	Low	No	Yes	No	No	No	No	No	-8,08	0,17
24	Low	No	Yes	No	No	No	No	No	-7,56	0,17
25	Low	No	Yes	No	No	No	No	No	-8,97	0,17
26	Low	No	Yes	No	No	No	No	No	-8,8	0,17
27	Low	No	Yes	No	No	No	No	No	-8,13	0,17
28	Low	No	Yes	No	No	No	No	No	-7,6	0,17
29	Low	No	Yes	No	No	No	No	No	-9,59	0,17
30	Low	No	Yes	No	No	No	No	No	-9,01	0,17
31	Low	No	Yes	No	No	No	No	No	-9,07	0,17
32	Low	No	Yes	No	No	No	No	No	-8,48	0,17
33	Low	No	Yes	No	No	No	No	No	-9,67	0,17
34	Low	No	Yes	No	No	No	No	No	-9,14	0,17
35	Low	No	Yes	No	No	No	No	No	-8.71	0,17
36	Low	No	Yes	No	No	No	No	No	-8.19	0,17

Table S3 – The computational predictions of pharmacokinetics parameters. The prediction of ADME parameters was performed with the freely accessible webtool SwissADME.[24]

B: Supplemental Figures

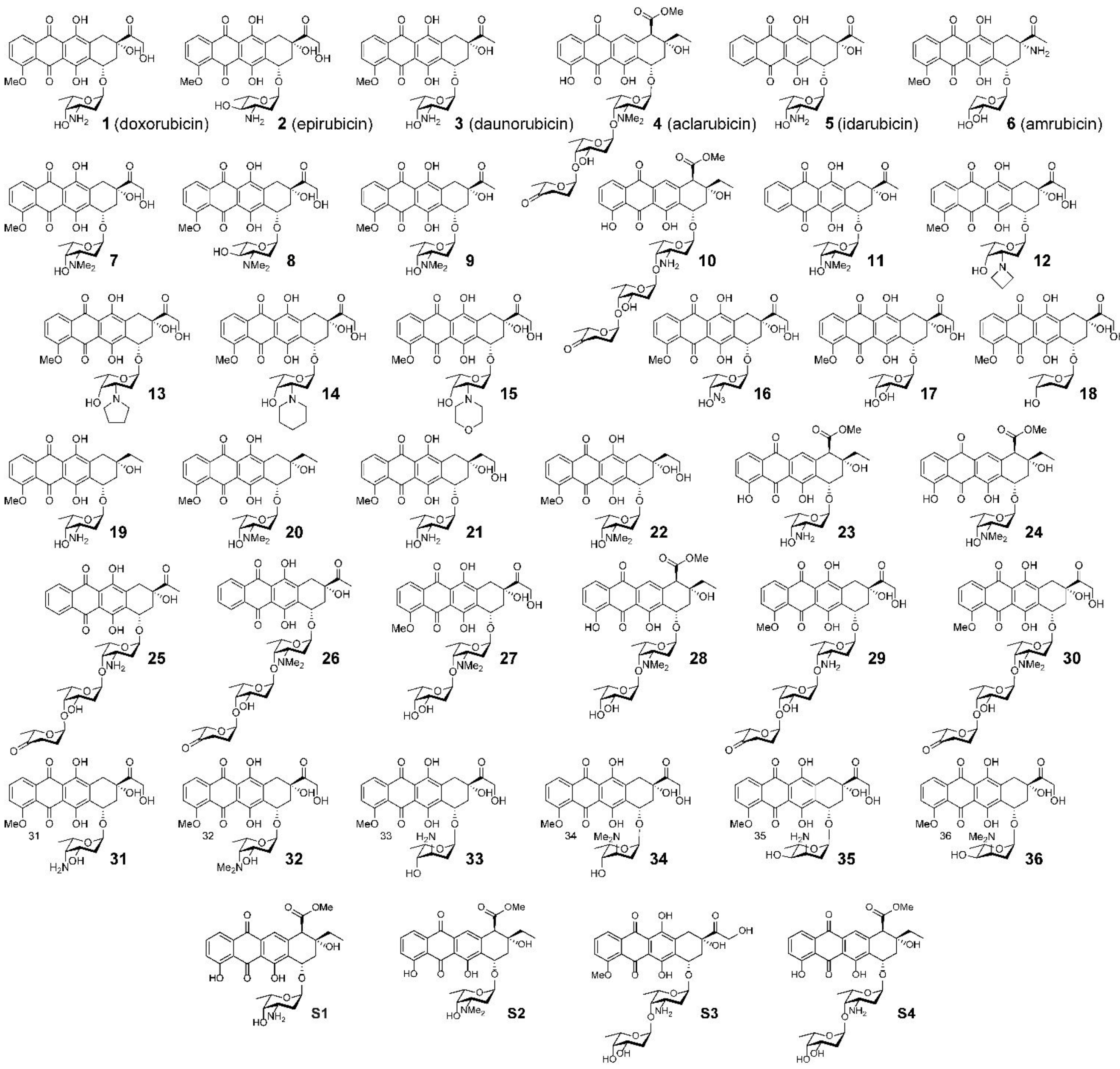

Figure S1 - Chemical structures of all compounds evaluated in this study; **1-36** and **S1-S4**.

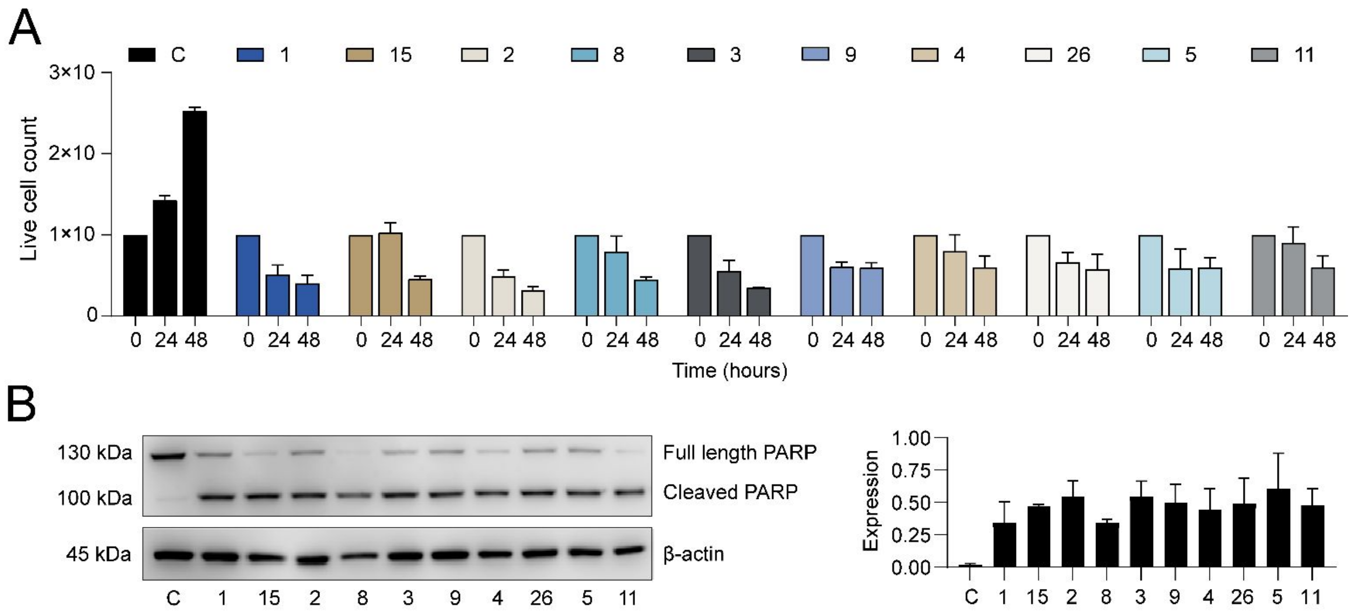

Figure S2 – Cell death in response to selected compounds 1-5, 8, 9, 11, 15, 26. Numbers correspond to the structures in Figure 1, C; unmanipulated control. (A) K562 wildtype cells were treated with 1 μM of the indicated compounds. The live/dead ratio was determined with DAPI staining and measured with flow-cytometry. (B) K562 wildtype cells were treated with 5 μM of the indicated compounds for 24 hours. PARP cleavage was examined by Western blot. Actin was used as a loading control, and molecular weight markers are indicated. Results are presented as mean ± SD of two independent experiments.

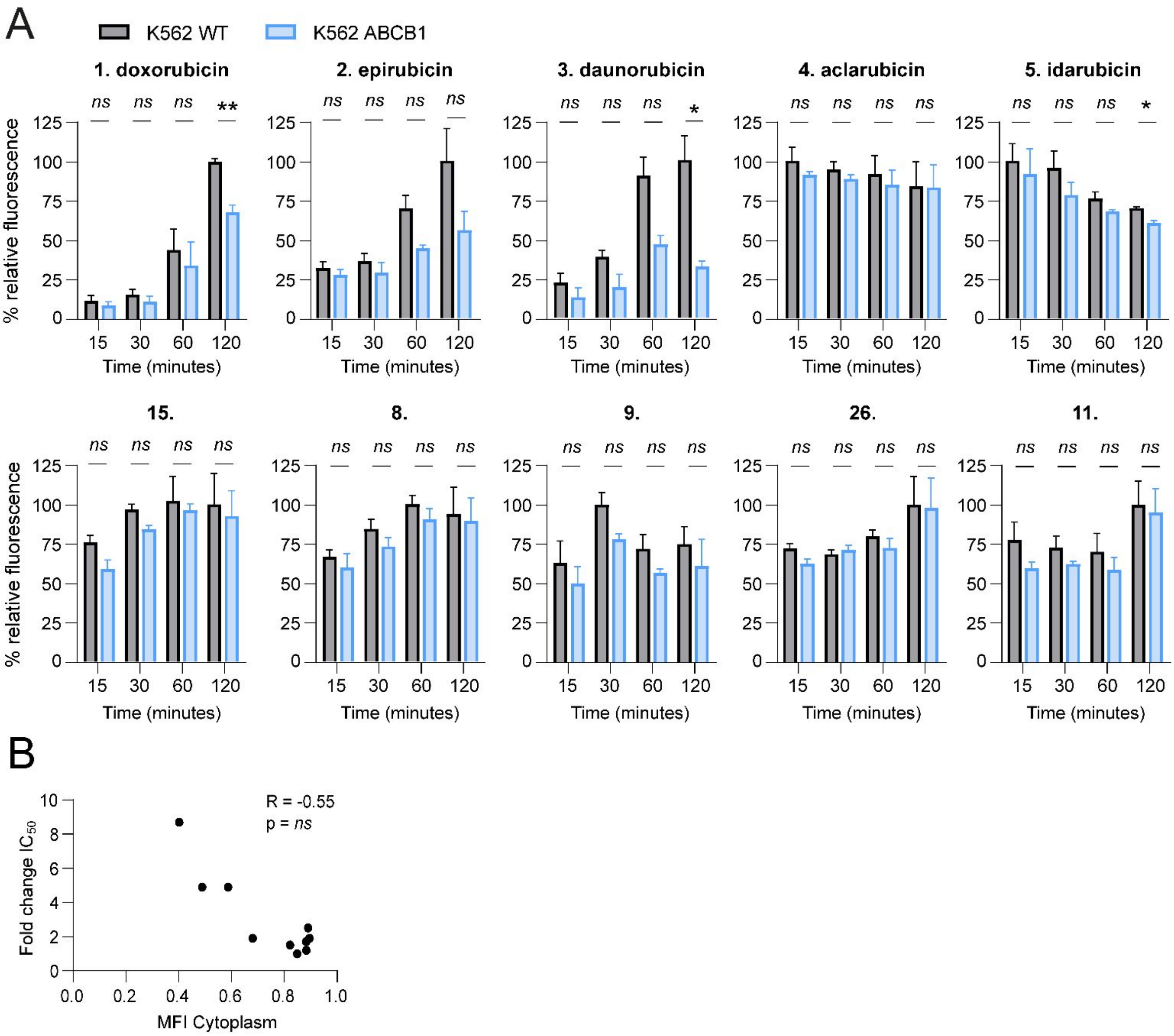

Figure S3 – Cytoplasmic accumulation of selected compounds **1**-**5**, **8**, **9**, **11**, **15**, **26**. Numbers correspond to the structures in Figure 1. (A) K562 wildtype and ABCB1 overexpressing cells were treated with 10 µM of the indicated compounds and subjected to fractionation. Mean fluorescence intensity in the cytoplasmic fraction was measured at a series of timepoints (15, 30, 60 and 120 minutes). Fluorescence was normalized to the largest signal. Two-way ANOVA; * $p<0.05$; ** $p<0.01$; *** $p<0.001$; ns, not significant. (B) Correlation between fold change IC_{50} and mean fluorescence intensity in cytoplasmic fraction.

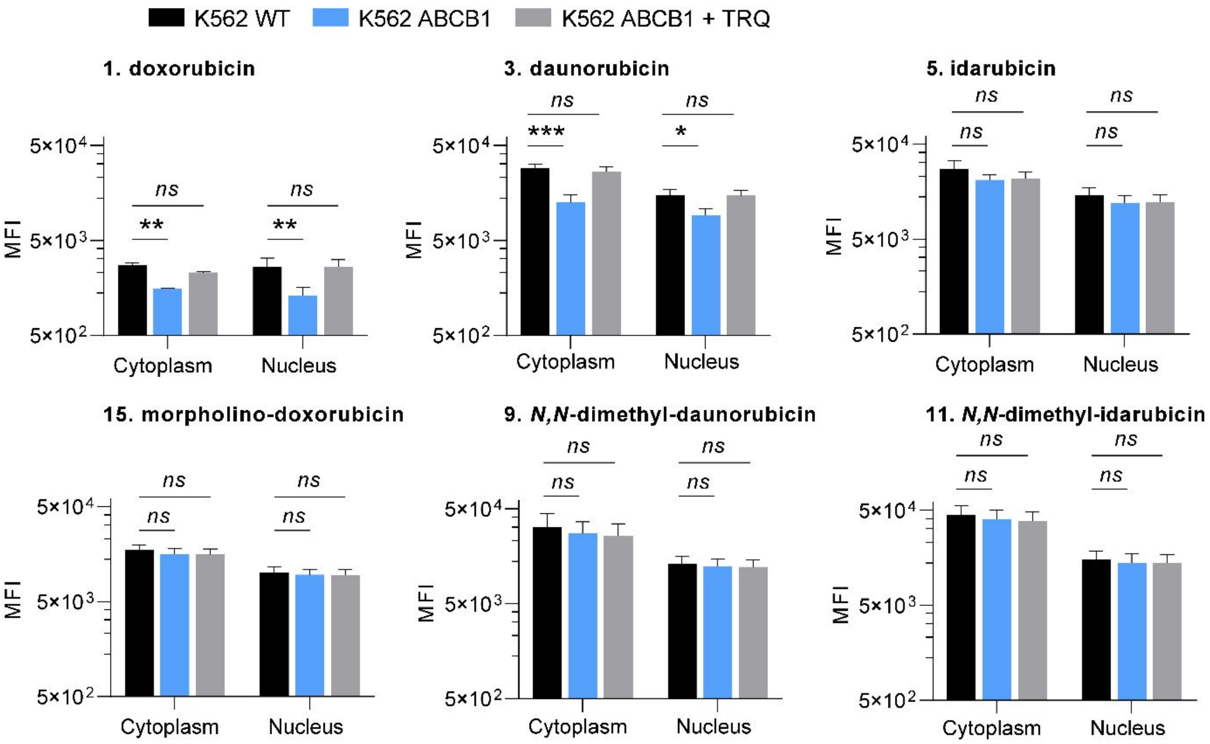

Figure S4 – Nuclear and cytoplasmic accumulation of selected compounds **1**, 3, **5**, **9**, **11**, **15**. Numbers correspond to the structures in Figure 1. K562 wildtype and ABCB1 overexpressing cells were treated with 10 µM of the indicated compounds, in the presence and absence of ABCB1-inhibitor Tariquidar. Mean fluorescent intensity was measured in cytoplasmic and nuclear fractions of K562 wildtype cells, ABCB1 overexpressing cells and ABCB1 overexpressing cells treated with Tariquidar (TRQ). Two-way ANOVA; * $p<0.05$; ** $p<0.01$; *** $p<0.001$; ns, not significant.

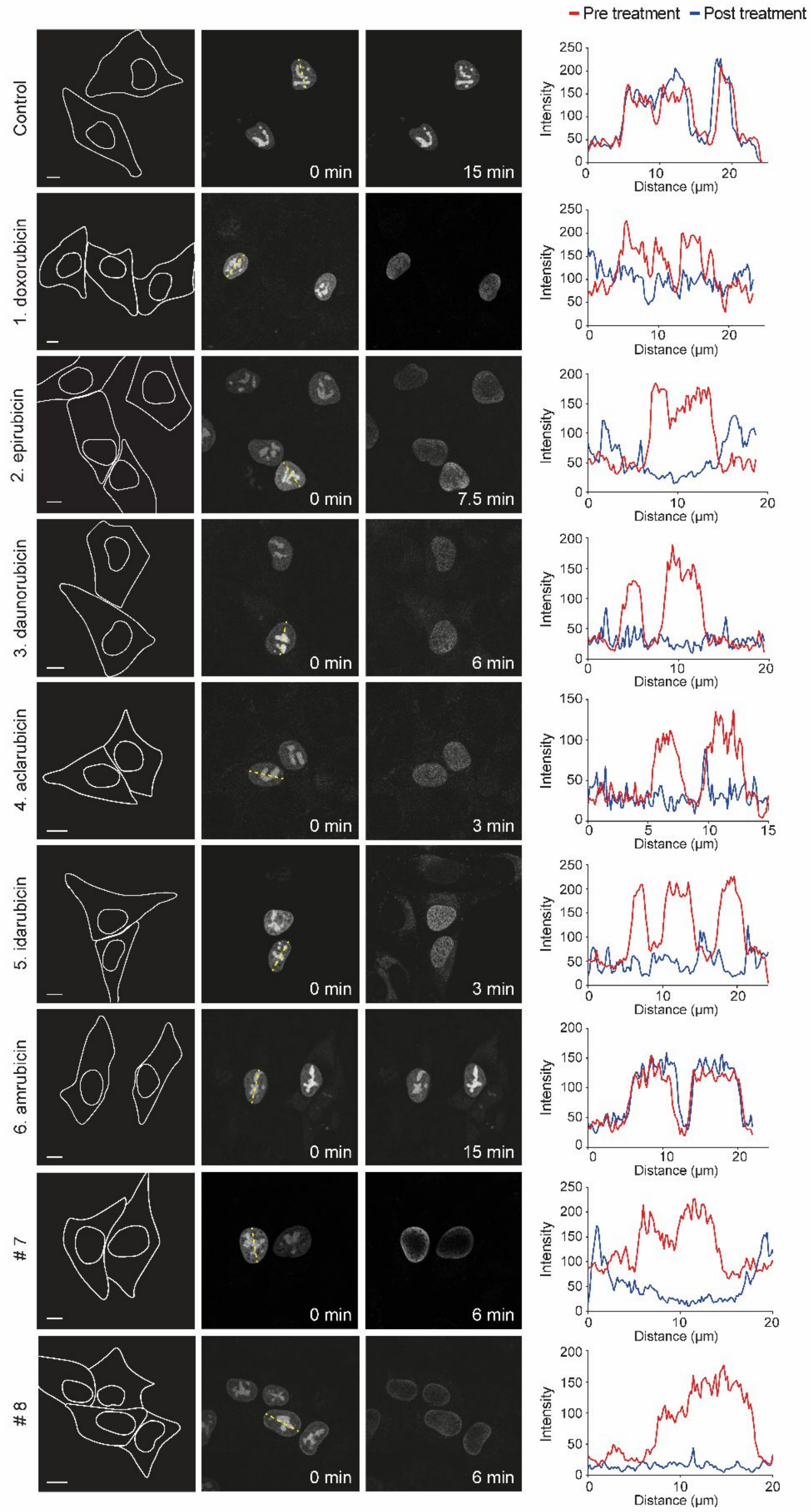

Figure S5 – Topoisomerase IIα relocalization for compounds **1**-**8**. Numbers correspond to the structures in Figure 1. Redistribution of GFP-TopoIIα transiently expressed in wildtype MelJuso. Cells were treated with 10 µM of indicated compounds and GFP-TopoIIα signal was measured over time. Scale bar 10µm. GFP signal was quantified pre- and post-treatment with the compounds and plotted as fluorescence over distance of dotted yellow line.

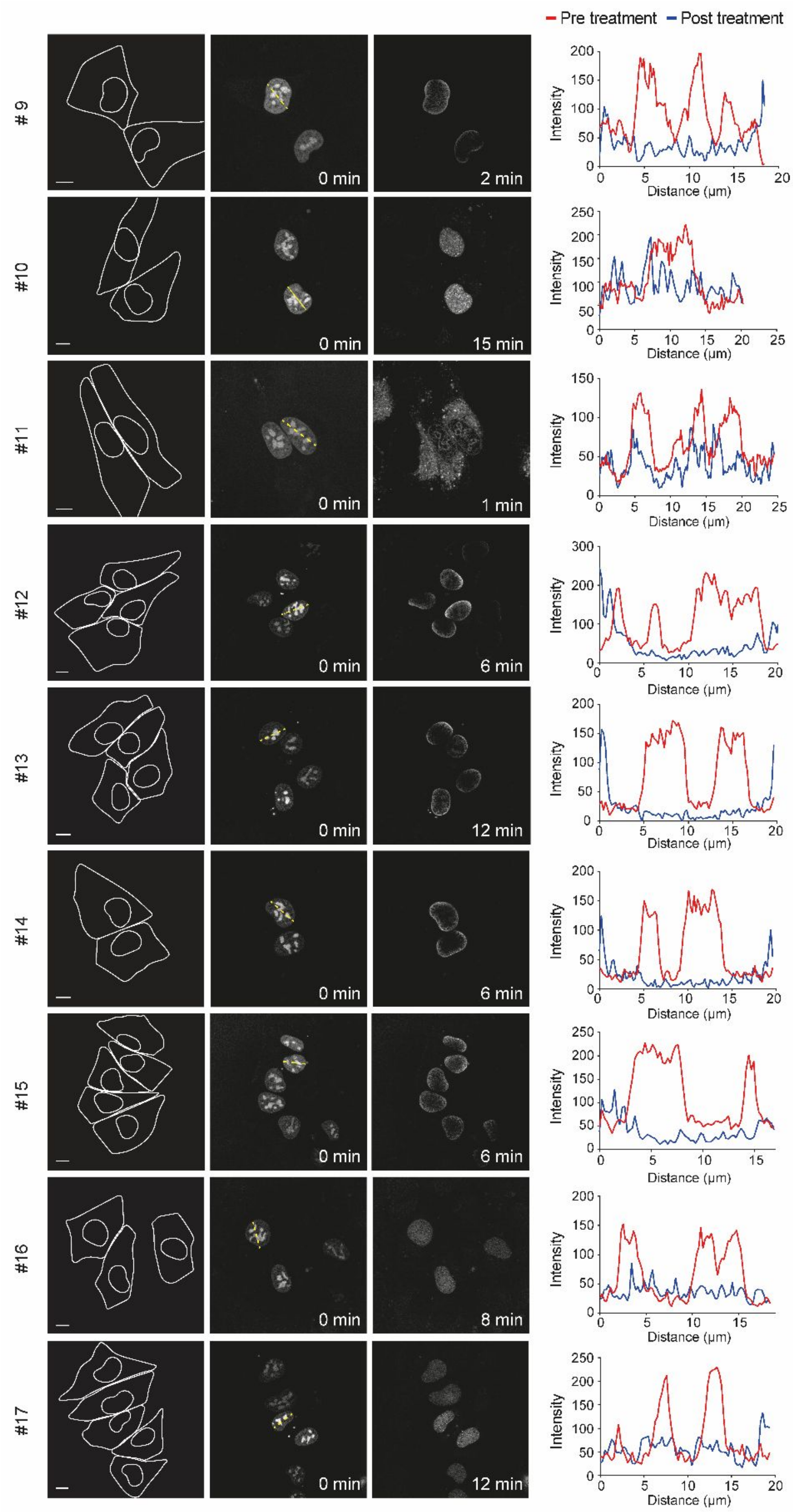

Figure S5 – Continued; Topoisomerase IIα relocalization for compounds **9**-**17**. Numbers correspond to the structures in Figure 1.

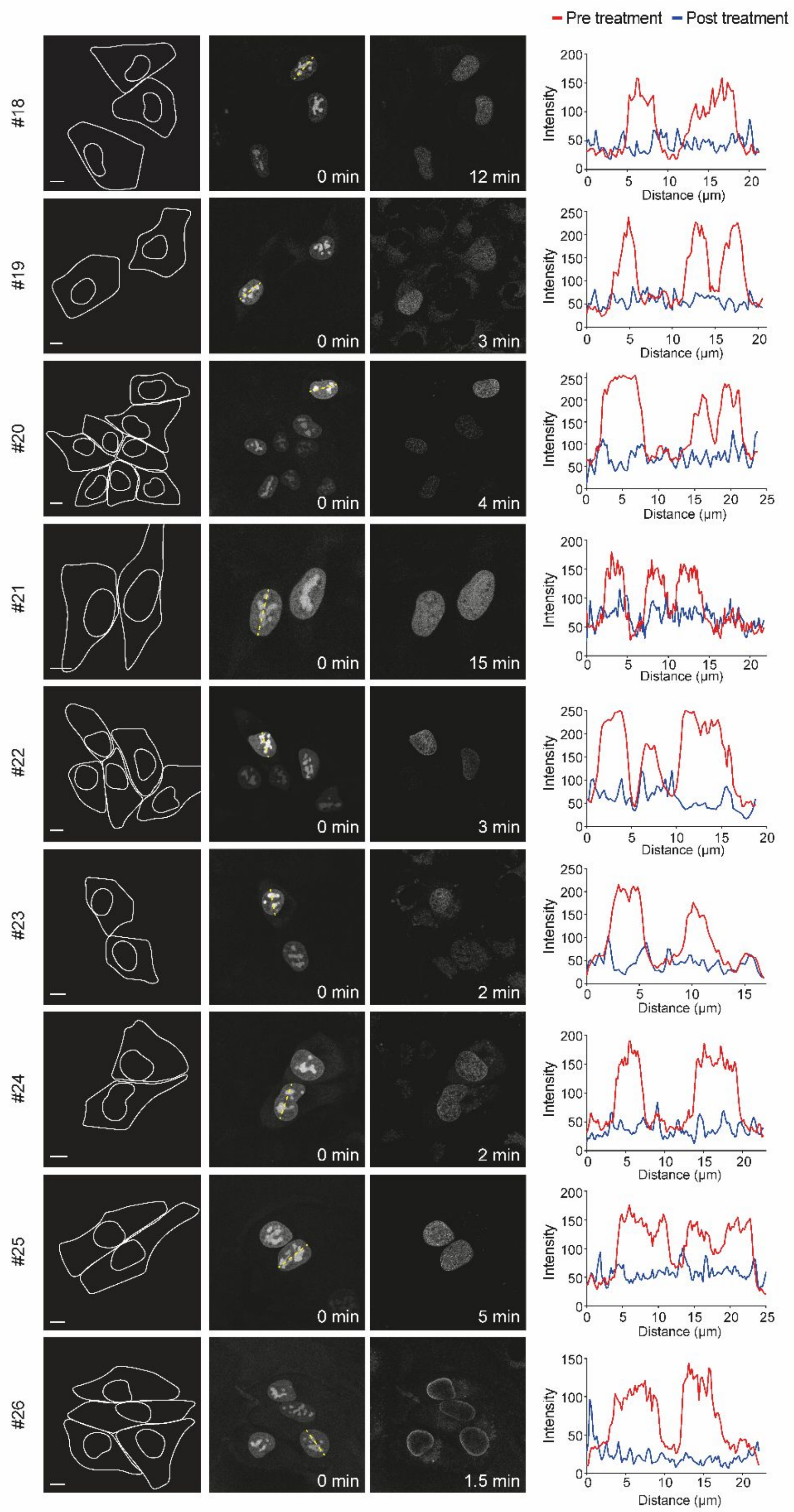

Figure S5 – Continued; Topoisomerase IIα relocalization for compounds **18**-**26**. Numbers correspond to the structures in Figure 1

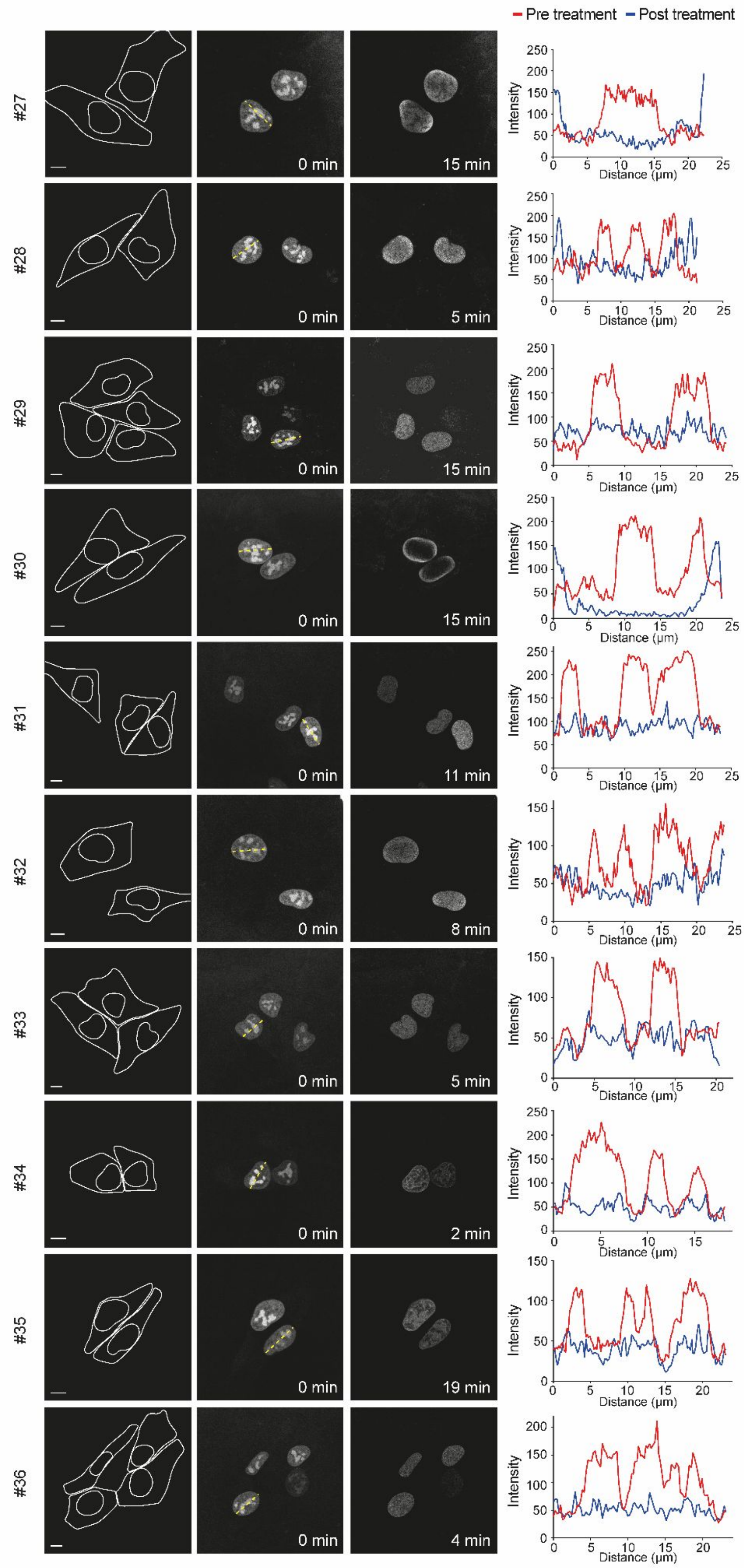

Figure S5 – Continued; Topoisomerase IIα relocalization for compounds **27**-**36**. Numbers correspond to the structures in Figure 1.

C: HPLC Traces

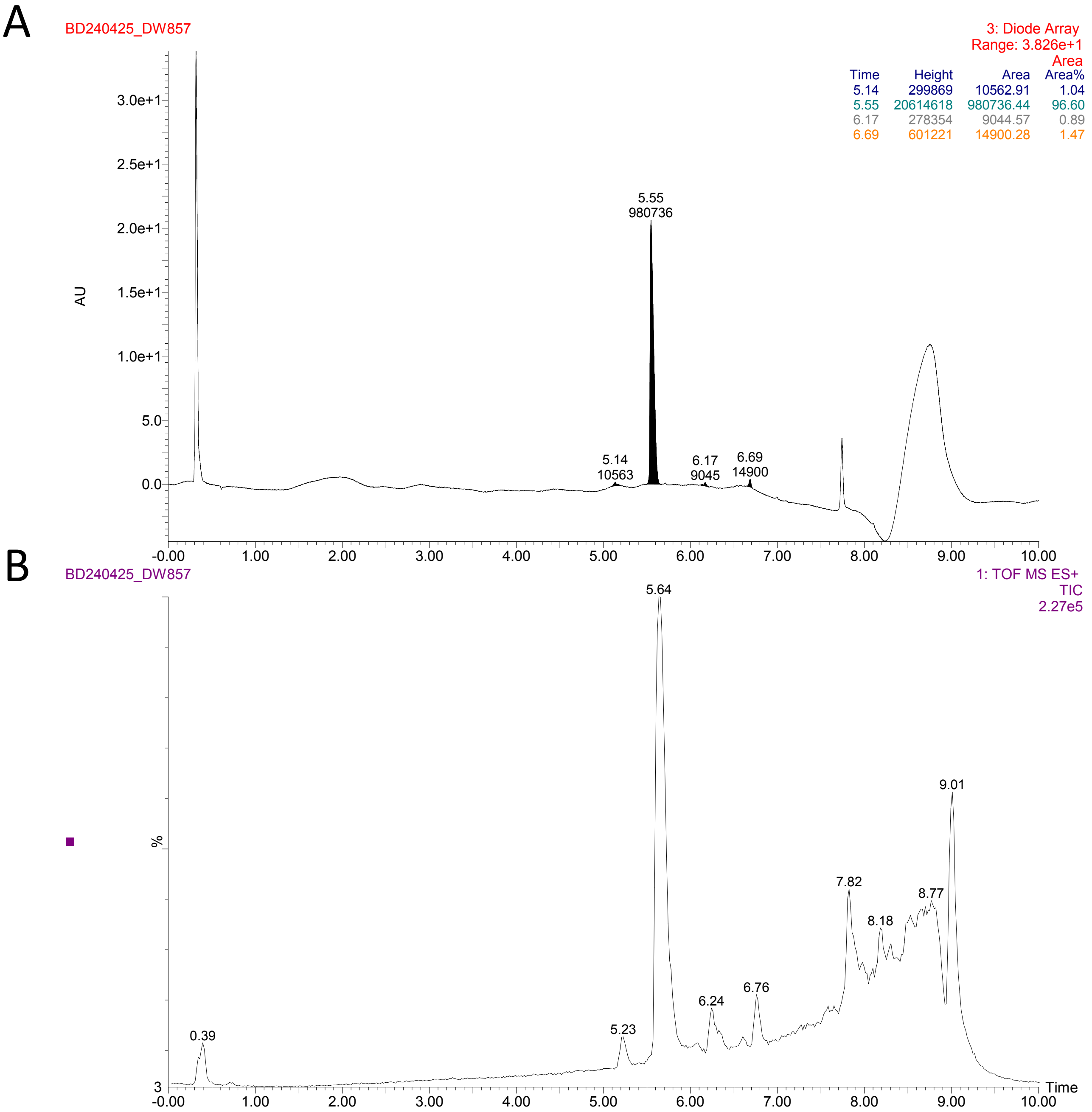

Figure S6 – Purity analysis of compound **11** using LCMS gradient. (A) UV trace was used for quantification. Main component peak at 5,55 min; product is 96,6% pure. (B) MS trace was used for identification.

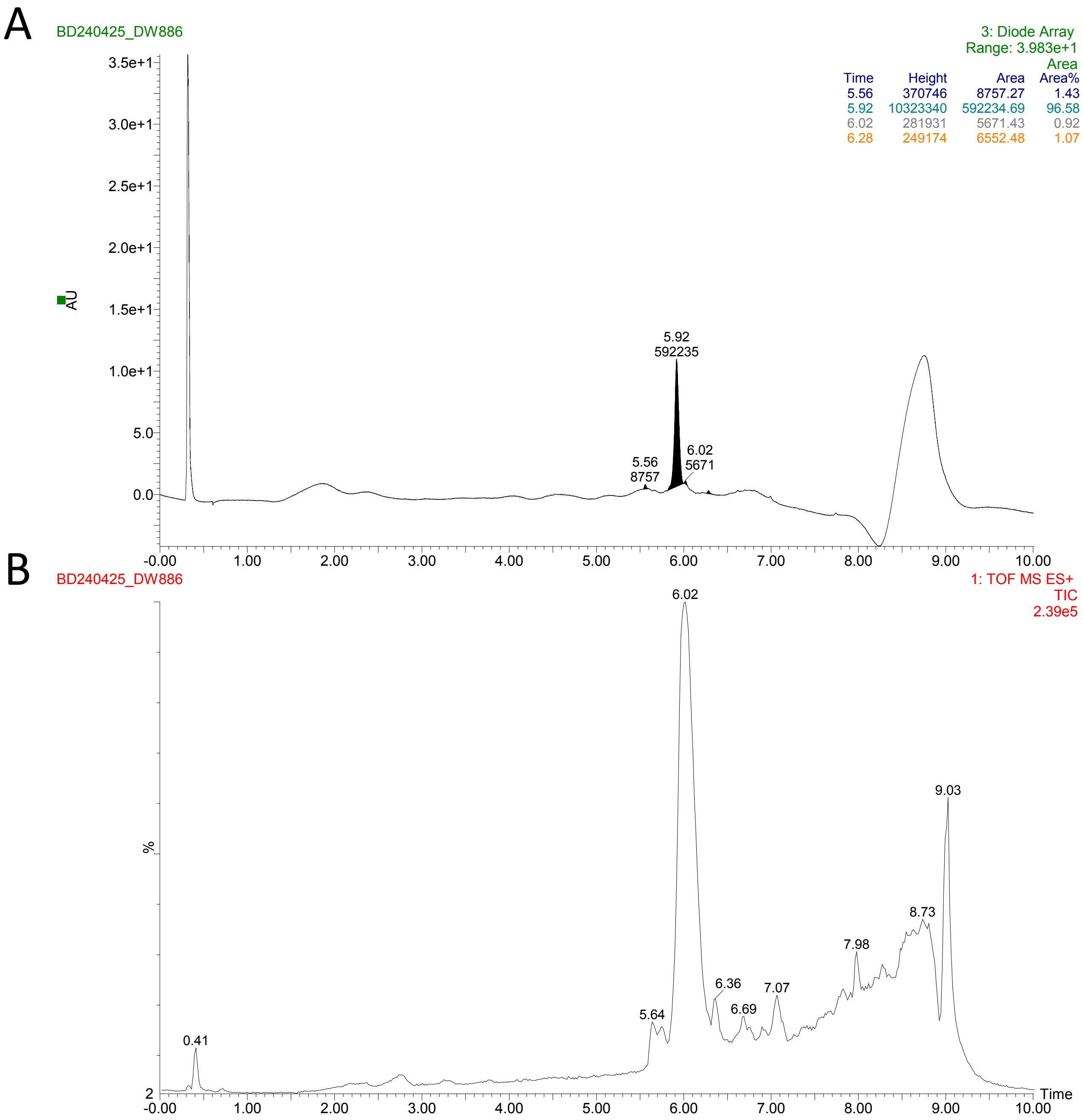

Figure S7 – Purity analysis of compound **26** using LCMS gradient. (A) UV trace was used for quantification. Main component peak at 5.92 min; product is 96,58% pure. (B) MS trace was used for the identification.
